# Supplementary material for: A high-resolution mRNA expression time course of embryonic development in zebrafish
Source: eLife. 2017 Nov 16;6:e30860. doi: 10.7554/eLife.30860 (PMC5690287; doi:10.7554/eLife.30860)
Supplement: Supplementary file 6. [file elife-30860-supp6.zip › biolayout-clusters-files/Cluster196-genes.html]

Cluster196


# Cluster196: Genes

| Ensembl ID | Gene Name | Chr | Start | End | Biotype |
| --- | --- | --- | --- | --- | --- |
| ENSDARG00000031795 | abcf1 | 19 | 30841600 | 30860324 | protein\_coding |
| ENSDARG00000045843 | apex1 | 4 | 5325418 | 5655206 | protein\_coding |
| ENSDARG00000053047 | eif2s2 | 6 | 49997128 | 50013273 | protein\_coding |
| ENSDARG00000056186 | eif5a2 | 2 | 26183559 | 26189703 | protein\_coding |
| ENSDARG00000077721 | knop1 | 1 | 54006003 | 54024014 | protein\_coding |
| ENSDARG00000091656 | lsm8 | 4 | 9535944 | 9538508 | protein\_coding |
| ENSDARG00000078214 | mybbp1a | 5 | 8208160 | 8260763 | protein\_coding |
